# Urinary Abscess

**Published:** 1856-03

**Authors:** C. B. Hutchins

**Affiliations:** Buffalo, N. Y.


BUFFALO MEDICAL JOURNAL

AND

MONTHLY REVIEW.

VOL. 11. MARCH, 1856. NO. 10.

### ORIGINAL COMMUNICATIONS.

ART. I.—*Urinary Abscess. A Dissertation read before the Medical Society of Erie County, January 8th*, 1856. By C. B. Hutchins, M. D., Buffalo, N. Y.

*Mr. President and Members of the Society:*

The gentleman who was selected as orator of the day, not being able to prepare himself for the occasion, the duty of addressing you devolved upon me. You would, undoubtedly, have been much better pleased had the person selected been able to appear before you, but as he could not fulfill the task assigned him, I shall claim your indulgence, while I strive, imperfectly as it may be, to fill his place.

I have concluded to bring before you at this time, the subject of urinary abscess. My reason for selecting it is, that my attention during the past year has been much occupied with this disease. It will be necessary to treat the subject in a somewhat discursive manner, since this disease is nearly always the result of another, therefore I shall follow no particular order in my remarks.

Urinary abscess usually manifests itself by a small, hard, circumscribed indolent tumor, not painful to the touch, and is generally found in the perineum, the scrotum, or penis. Occasionally it is seen in parts more distant from these regions. Charles Bell speaks of one in the groin, which was taken for a hernia. Other authors mention them in the thighs, the nates, the walls of the abdomen and in the lumbar regions.

When first discovered they are quite small, and they frequently remain so until they suppurate; but sometimes they increase to a large size, so that the whole perineum may be occupied by one of them. They are not always alone, occasionally we find several of them strung along the course of the urethra from the prostate to the glans penis.

They are produced by the urine slowly escaping from the urethra into the surrounding tissues. This process goes on so slowly that nature being warned has time to throw out lymph and confine the urine thus escaping in a small space. When the urine escapes rapidly without any such preparation, it is called infiltration of urine, and those cases of infiltration of urine which produce such frightful losses of tissue, are nearly always the result of urinary abscess; in what way will be hereafter explained.

Any thing which will cause an escape of the urine into the surrounding tissues is capable of producing urinary abscess. Among the causes are wounds, inflammations, and injuries of the urethra, strictures and the instruments used to cure them; the simple introduction of the catheter has been known to produce this disease. False routes made in attempts to dilate the urethra are a very frequent cause.

Perhaps they occur oftener in the following manner:

A person has one or more blenorrhagias which have lasted a long time. Sooner or later the stream of urine commences to grow smaller. At last the patient urinates with great difficulty, the urine coming away, drop by drop, with great straining, and requiring a long time to void it. In such a case the difficulty of urinating is caused by the formation of one or more strictures. Behind these the urethra gradually dilates. This dilatation goes on, the whole power of the bladder and abdominal muscles being put into force to void the urine, until finally one or more of the membranes of the urethra give way, when it escapes into the tissues, and urinary abscess is the result.

Another method of their production is, when an opening is made into the urethra, by a sharp cutting instrument, so as to destroy the parallelism of the edges of the wound, the urine escaping from it with difficulty finds its way into the surrounding parts.

One might infer that in all abrasions of the urethra abscess is the result. This is by no means the case; many times there are injuries of the urethra destroying its mucous coverings, and still there is no escape of the urine. As a rule it is necessary, in order that the urine shall escape from any abrasion, that its flow between that point and the external orifice should be interrupted; for how many times do we see the coats of the urethra destroyed by the application of caustic in the treatment of stricture, forward of the stricture, and still no abscess resulting.

Sometimes, however, abscesses are found along the course of the urethra, without any abrasion, which have the usual characters of urinary abscess. This variety is sometimes produced by the introduction of the catheter, particularly when it is done in a rude or harsh manner. They frequently occur also in the treatment of strictures by dilatation, when the size of the instrument is too rapidly increased. If in introducing an instrument into the urethra it is extremely sensitive, and if its repeated introduction, when done at a proper time, does not remove that sensibility, but on the other hand increases it, a persistance in its use will very frequently bring on this variety of abscess, and that this is the cause is proven, for, on abstaining from the use of it for a few days, the induration and swelling will disappear.

This variety of abscess without abrasion of the urethra, may also take place from a fall or a blow, or some violent inflammation of the urethra. It is said by some that in these cases that the urine passes by transudation; however it may be, there is a striking analogy between these abscesses and those found about the rectum, which have no communication with it, and still the pus discharged has the same fœtid odor as when the communication exists.

The same is the case in abscess of the right illiac fossa, when the pus is in contact with that portion of the coecum not covered with the peritoneum, and in urinary abscess without abrasion, the appearance and location of the tumor, its course and termination, and its contents, are precisely like those when the abrasion is evident, for if left to itself it will discharge into the urethra and afterward by the skin, thus producing fistula.

By keeping in view the anatomy of these regions we can often determine the portion of the urethra from which the urine escapes. If the point is above the middle aponeurosis of the perineum, in other words, in the membranous or prostatic portion, on account of the resistance met with from below, the urine in the first place makes its way toward the pelvis, then toward the ischro-rectal excavation, when abscesses in the vicinity of the anus develop themselves, and finally into the cellulo-adipose layer of tissues of the perineum and scrotum. When the urine escapes below this aponeurosis and forward of the commencement of the bulb, the abscess is usually found along the course of the urethra.

When those fearful injuries are produced from the urine finding its way p arts distant from the urethra, it will generally be found that the point of escape was in the prostatic or membranous portion; sometimes, however, inflammation destroys those barriers which would naturally control the direction of the urine, and on the other hand raises those which force it in directions so unexpected, that we are entirely unable to decide, except by a post-mortem, where the urine made its escape.

These considerations, perhaps, would cause us to act more promptly, for if we assure ourselves that the abscess is the result of an abrasion above the bulb, we cannot too soon make a passage for the urine externally.

Urinary abscess usually forms very slowly; it makes its appearance when near the skin as a small induration, which hardly attracts the notice of the patient. The tumor, if left to itself, gradually increases, and sometimes reaches a very large size; finally, however, nature reäcts and suppuration comes on. It may discharge by the rectum, the skin, or the urethra. The latter termination is much the most serious, for the urine then flows freely into the abscess, and there being no preparation by nature to restrain it, the organized lymph which had been previously thrown out to control it being in a condition of suppuration, it passes rapidly into the surrounding tissues, striking with gangrene everything with which it comes in contact. In such cases sometimes the perineum, the scrotum, and the tissues covering the pubis and thighs drop off, leaving an immense ulcer frightful to behold. It is well, when placed in such circumstances, to remember the words of Desault, who, in speaking of them, says:

"Practitioners who have never seen this disease, might be terrified at the extent of the ulcer resulting from the falling of the eschars. Sometimes the whole scrotum, the skin of the penis, that of the groin of the perineum, and the superior portion of the thighs, fall from gangrene, leaving the testicles suspended by their spermatic cords and floating in the midst of an enormous ulcer; it is almost impossible to conceive how a cicatrice can form on organs thus denuded, but nature has resources without bounds; she will glue the testicles with their cords to the subjacent parts, and drawing the skin from the circumference of the ulcer toward the center, will form for them a new envelop in form of a scrotum."

Chopart mentions a case when the fall of the eschars laid bare the prostate, a portion of the urethra, and the tunica vaginalis of the testicle, and still the patient recovered. From this we learn that after the most fearful losses of tissue from an infiltration of urine, we should never despair, but cling to our patient as long as there is life.

When they discharge into the rectum the case is generally prompt. The same remark may be made when they discharge by the skin.

These tumors sometimes commence very insidiously. Civiale gives a case, which I will cite, as it is a type of this variety of the disease:

"In the year 1837, a gentleman, 67 years of age, was operated on for stone; three years afterward there appeared a small tumor in the left buttock, which a skillful surgeon, who was consulted, considered hæmorrhoides, and as it gave no trouble did not examine it. A few weeks afterward the patient felt a good deal of pain and smarting whilst urinating. Then the tumor was examined, but it was found so small, hard and circumscribed, that there was not the slightest suspicion that it was a urinary abscess. The smarting, whilst urinating, was supposed to arise from a slight catarrh of the bladder, and an engorgement of the prostate; some simple calming and antiphlogistic remedies were prescribed. In a short time the patient became worse, and I was called to see him. I at once recognized that it was a case of urinary abscess, which had considerably increased during the last two days; fluctuation was evident, and a portion of the perineum was struck with mortification. The abscess was immediately opened. The incision, although deep, produced no pain. There came from it a large quantity of sanious fœtid pus. The patient was relieved, but in forty-eight hours it was necessary to again recur to the incision, as other points of fluctuation had declared themselves around the first abscess. From that time the mortification extended no farther, and he commenced to improve."

There are occasionally found along the course of the urethra, small abscesses, which, after suppurating, become lined with mucous membrane, thus forming a small pouch in which the urine sojourns. This is not emptied during micturition, unless pressed upon. This variety is frequently not discovered during life, and is only brought to light on a post-mortem examination.

The diagnosis of urinary abscess is usually easy, though sometimes very difficult. There is a variety of encysted tumor found along the course of the urethra, which is very similar to the tumor formed from the slow infiltration of urine. Their small size, their hardness and resemblance in all respects, can easily lead to mistakes of diagnosis. There is, however, one point of difference. The tumor produced by urinary abscess, usually forms more rapidly than the encysted tumor. If there is any difficulty of urinating, or any pain or smarting during micturation, or if there is any inflammation of the urethra, we may certainly conclude that we have urinary abscess. Also if a catheter, or bougie, has been introduced, and a small tumor should follow in a few days, we have reason to suspect it to be this variety of abscess. When, however, none of these causes are present, and the calibre of the urethra remains at its usual size, there is much difficulty in determining.

Perforation of the intestine sometimes takes place without any known disease, and in the same way perforation of the urethra, followed by urinary abscess, does take place, when there has been no apparent disease to explain it.

There are, also, abscesses in the vicinity of the prostate, produced by a morbid condition of the spermatic ducts, which have usually been considered as resulting from the infiltration of urine; this disease is frequently unobserved until after death, and there is yet much to be learned about it.

When, however, a tumor is found along the course of the urethra, and in connection with it a burning, smarting pain on voiding the urine; and if in addition to this there is a stricture of the urethra, there can be but little doubt of its character. We may safely conclude that it is a case of urinary abscess.

There are two indications of treatment in this disease: First, to give exit to the contents of the tumor; secondly, to control the urine so as to prevent its passage into the abscess and tissues surrounding. The first indication is carried out by making a free incision into the tumor, and this should be very deep; and if it does not give free exit to the contents others should be made until this result is obtained. A person inexperienced in these abscesses might be astonished at the depth to which it is necessary to carry the knife in order to permit the pus to escape; also at the quantity of pus which comes from them. A small tumor is discharged. Here we only feel a small portion of the tumor, as the rest is deep seated and beyond our reach.

If the pus has a free exit by the skin, there is but little danger that it will find its way into the urethra, for, as the opening by which the urine escaped from the urethra is usually very small, it will soon close; but if the abscess discharges into the urethra we are almost certain to have a fistula to combat in addition to the abscess.

Should a tumor arise along the course of the urethra during its dilatation for the cure of stricture, the instrument should be laid aside for a few days and resumed with great caution, as time and patience will overcome the most irritable urethras.

When there is already an opening into the urethra and by the skin, the urine must invariably be drawn off by the catheter, else there will be new abscesses constantly forming around the first.

I have had under my care during the past year, an interesting case of this disease which I will give you in as few words as possible:

About the middle of November, 1854, I was called to see a man about 35 years of age, not of very robust constitution; complexion pale, with sandy hair and blue eyes. His occupation was following the canal. He had been troubled several years with a difficulty of urinating. Previous to this he had contracted several blenorrhagias, and for a few years past there had been a constant discharge from the urethra. He had also been afflicted with numerous chancres and buboes, the latter of which had suppurated, leaving large cicatrices in the groin. He had been under treatment several times for his difficulty of urinating, which had consisted entirely of internal remedies and injections in the penis, except at one time when bougies had been used for a few days in Memphis, Tenn. When I found him there was a large abscess occupying the scrotum and penis, and on the right and under side of the penis just forward of the scrotum, there was a fistulous opening from which there was a constant discharge of pus, and on voiding his urine the largest part passed by this opening. The health of the patient had suffered considerably, as in addition he had a quotidian ague. On inserting a small bougie it passed readily until it reached that portion of the urethra just in front of the fistula, beyond which it would not penetrate, nor could I pass that point with my smallest bougie; I immediately commenced dilating the urethra.

A week or ten days afterward, the patient called my attention to a small tumor in the perineum, which was a new abscess forming. I was about to lay it open when he passed out of my hands into the care of another practitioner (who I see is present to-day as a spectator.) This was about the first week of December. I saw nothing more of him until the 26th of the same month, when I was again called to treat him.

On examination I found that the abscess in the perineum had opened. There was a good deal of infiltration of urine, so much so that I feared that the scrotum would slough away with portions of the perineum. He was now very much emaciated, and so debilitated as scarcely to be able to turn himself in bed. The appetite was gone and the pulse feeble and frequent. There was so much cough and expectoration that I feared tuberculosis. From the fistulæ there was a constant discharge of fœtid pus mixed with urine. I immediately made four incisions into the perineum, which reached forward to the scrotum, and passed my smallest flexible catheter into the bladder. This was a very difficult task and required long continued and assiduous efforts. I found but little urine in the bladder. The catheter was left in situ with a plug in the end, and the patient directed to draw off his urine every three hours. On the following day I found that the catheter had been well borne, and on withdrawing it succeeded in replacing it by a size larger. Every two or three days I was able to increase the size of the catheter until

No. 6 was reached, when I was arrested by the small size of the orifice of the urethra. This I found necessary to incise several times. The smallness of this orifice was caused by the existence of previous chancres at the extremity of the penis. Each morning the bladder was injected with tepid water, which was drawn off by the catheter, then re-injecting it and drawing out the instrument the patient was directed to force off the contents of the bladder by the urethra, thereby cleansing the diseased tissues, after which I threw into the penis with a small syringe, a mild solution of the sulphate of zinc, until it passed freely by both fistulas. Frequently on withdrawing the catheter I was obliged to pass my finger into the rectum in order to reintroduce it. I then to a certain extent could measure the deep-seated portion of the abscess. The end of the catheter was distinctly felt in front of the prostate, there being nothing but the walls of the intestine intervening. It extended to the ischium on both sides and from the prostate to the bulb. The prostate had a very spongy feeling, and seemed slightly enlarged.

The internal treatment was quinine and the tinct. of iron; also ale and wine, with generous diet.

During the treatment there was twice engorgement of the testicles, which compelled me to suspend the use of the bougies for a few days; but the urine was invariably drawn off by the catheter. There were two very unyielding callous points in the urethra, one of which would nearly sever the bougie after it had been worn for a few days. These gave way very slowly, and I feared that it would be necessary to use the scarificator, but the bougies finally triumphed.

Under this treatment the patient steadily improved. His appetite returned and he rapidly gained flesh. The induration of the perineum, scrotum and penis, rapidly disappeared. The fistulas gradually closed. After he had ceased wearing the bougies the urine was drawn off by the catheter. During the month of March I ceased visiting him, and rarely saw him afterward. During the summer he contracted another blenorrhagia, which was cured in a few days without producing any bad effects. The last time I saw him, which was in July, he told me that he had laid aside the bougies and that the urine all flowed by the urethra. I wished to cauterize the fistulas long before this, but I could not get him to my office to submit to it.

The variety of flexible catheter used in the commencement of the treatment of this case, is sometimes invaluable. They were bent in the form of the usual silver catheter, and held that form. I found it impossible to pass a silver catheter, for it would go beyond the stricture on reaching the cavern in front of the prostate, where the urethra was floating in pus, and its coats very much softened, and it would have been very difficult to reach the bladder without making a false route.

I made one mistake in the treatment of this case. The incisions were too few and not deep enough. Civiale says that this is generally the fault with young practitioners in this disease.

I am convinced if the incisions had been much more extensive the cure would have been hastened by several weeks.

